# Autosplenectomy in a Patient With Autoimmune Polyglandular Syndrome Type 2 (APS‐2)

**DOI:** 10.1155/crie/6610410

**Published:** 2026-01-28

**Authors:** Luqman S. Fauzi, Airin Jyoty, Yashwin Sredharan, Manohara Kenchaiah

**Affiliations:** ^1^ Department of Diabetes and Endocrinology, Northampton General Hospital, Northampton, UK, nhs.uk

**Keywords:** autoimmune polyglandular syndrome type 2 (APS-2), autosplenectomy, hyposplenism/asplenia, overwhelming post-splenectomy infection (OPSI), progressive splenic atrophy

## Abstract

Autoimmune glandular syndrome type 2 is a complex genetic condition where a triad of endocrinopathies is involved, namely, Addison’s disease, type 1 diabetes, and/or autoimmune thyroid disorder. The disease predisposes one to a variety of other autoimmune associations. Here, we report a rare presentation of a patient with autoimmune polyglandular syndrome type 2 (APS‐2) presenting with a 7‐year history of progressive splenic atrophy causing functional hyposplenism that ultimately progressed to anatomical asplenia (autosplenectomy) as demonstrated in the serial imaging. We postulate that the underlying cause of this presentation is also of autoimmune nature. Unlike APS‐1, which has been linked to hyposplenism, this is the first reported case of APS‐2 with similar splenic involvement. Splenic hypofunction can increase susceptibility to encapsulated bacterial infection, with overwhelming postsplenectomy infection (OPSI) being a significant threat. It is crucial that clinicians recognize the importance of providing guidance on vaccinations, antibiotic chemoprophylaxis, and patient education for individuals with asplenia or hyposplenism. If patients with APS can experience progressive splenic atrophy, we suggest long‐term follow‐up with splenic function assessment. It is yet unclear whether preemptive screening with pitted red cell count has any clinical impact in this group of patients.

## 1. Introduction

Asplenia refers to the absence of effective splenic function which can either be congenital or acquired. It can be further categorized into anatomical asplenia in which the spleen is physically absent or functional asplenia (hyposplenism), where splenic tissue is present but immune function is impaired [1]. In this report, we use hyposplenism to denote impaired splenic function, anatomic asplenia to describe complete absence of splenic tissue, and autosplenectomy to describe a progressive destructive process resulting in anatomical asplenia.

As the spleen is a secondary lymphoid organ involved in the regulation of immune responses, both anatomical asplenia and hyposplenism significantly increase susceptibility to severe and invasive infections, particularly those caused by encapsulated bacteria. Notable pathogens include *Haemophilus influenzae* type b, *Streptococcus pneumoniae*, and *Neisseria meningitidis*.

Autoimmune polyglandular syndromes (APSs) are clinical conditions characterized by multiple endocrinopathies as a result of loss of immune tolerance [2]. Classically, it can be classified into APS 1‐4. Asplenia and APS‐1 were first noted in 1968 [3]. Since then, several cohort studies have identified modest association between asplenia and APS‐1 [4–6]. Autoimmune polyglandular syndrome type 2 (APS‐2) is a more frequent syndrome than APS‐1. To be diagnosed with APS‐2, a patient must have at least two of the three endocrinopathies: Addison’s disease, type 1 diabetes, and autoimmune thyroid disease. Our patient exhibits all three of these endocrinopathies, suggesting an underlying diagnosis of APS‐2. In contrast to the well‐documented reports of asplenia in APS‐1, the existing literature appears to lack substantial evidence of an association between APS‐2 and splenic hypofunction. Here, we report a case of a patient with a diagnosis of APS‐2 who developed progressive splenic atrophy leading to autosplenectomy.

### 1.1. Case Presentation

A 56‐year‐old Caucasian lady presented to the emergency department with a 2‐day history of abdominal pain associated with reduced mobility and urinary retention. Her biochemical profile suggested an acute prerenal injury (eGFR 15 mL/min and creatinine 302 µmol/L). Her urine microscopy demonstrated >200/µL of white cells associated with significantly raised serum inflammatory markers. She was treated with an underlying diagnosis of pyelonephritis with a 9‐day course of intravenous antibiotic Meropenem, followed by another change to a combined intravenous regimen Gentamicin and Cefuroxime due to unresolving inflammatory markers. Considering persistently raised inflammatory markers, a computated tomography (CT) imaging of her abdomen was obtained which showed evidence of right perirenal fat stranding in keeping with acute pyelonephritis. Following the switch, inflammatory markers declined, and renal function improved to baseline (eGFR ~85 mL/min) prior to discharge; there was no clinical recurrence of pyelonephritis on follow‐up.

**Figure 1:**
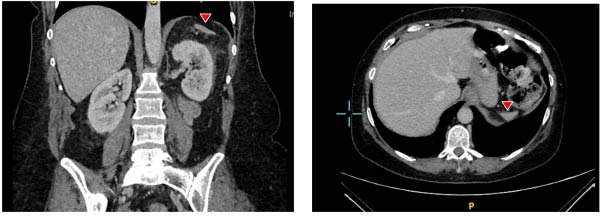
Sagittal CT image showing a markedly atrophic spleen in the left subdiaphragmatic region (red arrow). Retrospective review of a coronal CT scan performed 1 year earlier demonstrated similar splenic atrophy.

Incidentally, the CT scan of the abdomen also revealed a markedly atrophic spleen, a new finding compared with an abdominal ultrasound 5 years earlier that had reported a normal spleen. On retrospective review of imaging from 1 year prior to this admission, a CT scan was found to have shown a similarly atrophic spleen, although this has not been commented on in the original report. CT angiography of large abdominal vessel was not done. Following discharge, she was referred to hematology, where peripheral blood film demonstrated Howell‐Jolly bodies and target cells, confirming hyposplenism. She remained under regular follow‐up, and subsequent imaging later confirmed progression to complete splenic atrophy (as detailed in the follow‐up section) (Figure 1).

Her past medical history includes type 1 diabetes mellitus of 31 years’ duration prior to this presentation for which she is on hybrid closed loop insulin pump delivery. She was also diagnosed with Addison’s disease in 2001 and is treated with oral hydrocortisone regimen of 10 mg morning/5 mg midday/5 mg evening and oral fludrocortisone 50 µg daily. Additionally, she has an underlying diagnosis of autoimmune hypothyroidism for which she is on oral 100 µg levothyroxine daily. Her other medical history includes a recurrent urinary tract infection with *E. coli*, exceeding seven instances since 2008. Additionally, she has experienced one episode of meningitis and a sacral wound infection caused by *E. coli*. There was no personal history or family history of sickle cell anemia, no known history of abdominal trauma or substance abuse. Her daughter has also been diagnosed with autoimmune hypothyroidism. There are no other known autoimmune or endocrinological diseases among her close family members.

### 1.2. Diagnostic Assessment

Her laboratory work‐up on admission is consistent with an acute inflammatory response as a result from pyelonephritis. This is presented in Table 1.

**Table 1:** Laboratory results at admission and immunological investigations.

Parameters	Initial presentation	Normal values
Hemoglobin	111	120–150 g/L
White cell count	14.6	4.0–10.0 × 10^9^/L
Platelet count	244	150–400 × 10^9^/L
Neutrophils	13.33	1.8–7.4 × 10^9^/L
C‐reactive protein (CRP)	303	0–5 mg/L
Estimated eGFR	15	>90 mL/min
Sodium	126	133–146 mmol/L
Potassium	4.3	3.5–5.3 mmol/L
Creatinine	302	60–120 µmol/L
Urea	13.5	3.3–6.7 mmol/L
Albumin	34	35–50 g/L
Corrected calcium	2.36	2.20–2.60 mmol/L
Inorganic phosphate	1.46	0.8–1.5 mmol L
Total bilirubin	5	0–21 µmol/L
Alkaline phosphatase	89	30–130 IU/L
Alanine transaminase	21	5–33 IU/L
HbA1c	61	≤48 mmol/mol

Immunological and blood film findings		
Thyroid antibody	Thyroid peroxidase (TPO)	>1300 U/mL (positive)
Celiac screen	Tissue transglutaminase IgA	0.5 U/mL (negative)
	IgA antibody	1.8 g/L
Rheumatology screen	MPO ANCA PR3 ANCA Extractable nuclear antigen (ENA) Double‐stranded DNA (ds‐DNA)	<0.2 IU/mL (negative) 0.4 IU/mL (negative) Negative Negative
Autoimmune hepatitis screen	Antimitochondrial antibody (AMA) Antismooth muscle antibody (ASMA) Antiliver/kidney microsomal antibody (anti‐LKM1)	Negative Negative Negative
Blood film	Target cells and Howell‐Jolly bodies seen	

### 1.3. Treatment

She was treated under the urology team with intravenous antibiotics as per hospital guideline for pyelonephritis, and her hydrocortisone dose was doubled throughout her admission. A week following her discharge, she developed a swollen calf and was diagnosed with deep venous thrombosis (DVT) in one of her paired peroneal veins. She commenced on the standard oral anticoagulant treatment. It was uncertain if her DVT was due to an inpatient stay or may be associated with a state of hyposplenism.

### 1.4. Outcome and Follow‐Up

**Figure 2:**
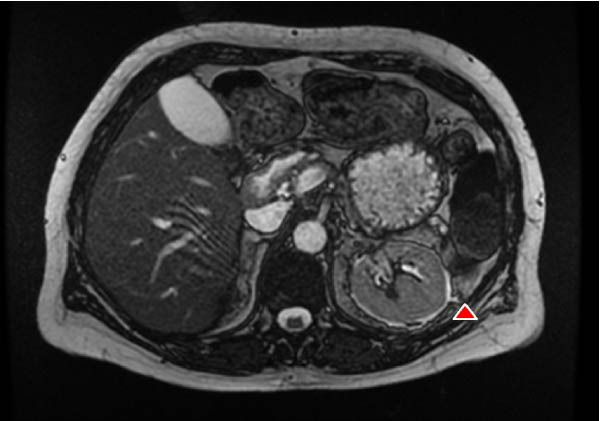
Follow‐up imaging at 2 years, MRCP confirmed anatomical asplenia (no splenic tissue visualized), consistent with autosplenectomy.

The patient remained under regular monitoring by the diabetes and endocrinology team to manage her APS‐2. A hematology review was conducted for her hyposplenism, leading to the initiation of penicillin V prophylaxis at 250 mg twice daily, along with recommendations for additional vaccinations [7]. A subsequent magnetic resonance cholangiopancreatography scan, performed 2 years later, revealed an atrophic pancreas in keeping with her known history of type 1 diabetes, moderate hydronephrosis, and anatomical asplenia (Figure 2).

## 2. Discussion

APS‐2, also known as Schmidt’s syndrome, is a complex genetic condition influenced by specific HLA haplotypes that predispose individuals to multiple autoimmune conditions [8]. APS‐2 is the most common type among the APSs. Its primary component is Addison’s disease, occurring in combination with either type 1 diabetes mellitus or autoimmune thyroid disease. Less frequent associations include myasthenia gravis, stiff man syndrome, and alopecia [8].

Asplenia may develop gradually with several mechanisms—progressive splenic atrophy, architectural distortion, or via disruption of B cell microenvironment [1]. However, these mechanisms are not exclusive to autoimmunity and are also seen in other disorders such as hematological disease (e.g., sickle cell disease), celiac disease, and infections. Notably, anatomical asplenia has also been described in systemic lupus erythematous (SLE) and autoimmune hepatitis (AIH) [9, 10]. Our patient did not present with symptoms, signs, or biochemical evidence to suggest that her asplenia was due to other disorders mentioned above.

In APS specifically, splenic dysfunction has not been previously considered a direct manifestation; however, similar to how APS can cause endocrinopathies through autoimmune destruction, our patient’s course suggests a similar autoimmune‐mediated splenic injury. In APS‐1, hyposplenism worsen with age, supporting an autoimmune etiology [11]. This is similar to the midlife onset and progression in the patient’s case.

Retrospectively, additional testing could have been done earlier to characterize splenic function. If hyposplenism was confirmed, earlier preventative measures could have been deployed. Pitted red cell count is regarded as the gold‐standard, cost effective test for diagnosing hyposplenism [1]. It quantifies circulating erythrocytes with surface pits that are normally cleared by the spleen. Although the presence of Howell‐Jolly bodies on blood film has been used as a marker, it is insensitive for mild hyposplenism. Radioisotope scans are no longer favorable due to radiation exposure and high cost [1].

There is no clear evidence that splenic atrophy increases thromboembolic risk. Some studies have associated splenectomy with thrombosis, particularly in the population with hematological disorders [12, 13]. In this patient, the DVT is likely to be related to recent hospitalization rather than hyposplenism per se. Her recurrent UTI and pyelonephritis, however, may relate to impaired splenic function.

Loss of splenic function markedly increases susceptibility to encapsulated bacteria and overwhelming postsplenectomy infection (OPSI) as a result of depletion of IgM memory B cells (1). OPSI can present as a rapidly progressive illness leading to fulminant sepsis and septic shock. Although OPSI is uncommon (≈3%–7%), the mortality rate may approach 70% [14, 15], with the risk persisting beyond 5 years postsplenectomy [16].

Therefore, clinicians should advocate for preventative care. Prophylactic antibiotics and vaccinations should be provided, including annual influenza immunization, a 5‐yearly booster dose of pneumococcal polysaccharide vaccine (PPV), and vaccination against meningococcal groups, A, C, W, Y, and B, as outlined in Chapter 7 of the UK Green Book [7]. The British Society of Hematology also encourages patient education, particularly regarding the risks of travel to regions with high malaria prevalence or exposure to zoonotic infections [17].

For patients with APS who have recurrent infections or hematological clues to hyposplenism, clinicians should assess splenic function early and implement prophylaxis promptly. Given the clinical trajectory in this case, screening for hyposplenism in APS‐2 using pitted red cell may be reasonable, although its impact on outcomes requires further study.

Although APS‐1 has been documented as a cause of hyposplenism and anatomical asplenia [1, 18], only a few cases have linked splenic dysfunction to APS‐2. Atquet et al. [19] reported a 37‐year‐old woman with APS‐2 and unexplained thrombocytosis, later diagnosed with hyposplenism likely due to autoimmune splenic atrophy. In 2017, another case described a 69‐year‐old woman with APS‐2 in whom anatomic asplenia was incidentally identified on imaging, confirmed by absence of splenic uptake on radionuclide scanning [20]. Our case aligns with these two reports but demonstrated a progressive transition from hyposplenism (with residual splenic tissue) to complete anatomical asplenia over serial imaging, consistent with autosplenectomy.

This report expands the current knowledge of autoimmune phenotype of APS‐2 to include asplenia. If APS patients are indeed at increased risk of splenic impairment, screening for hyposplenism might be a notion worth exploring.

## Funding

No funding was received for this manuscript.

## Consent

All the patients allowed personal data processing, and informed consent was obtained from all individual participants included in the study.

## Conflicts of Interest

The authors declare no conflicts of interest.

## Data Availability

The data that support the findings of this study are available from the corresponding author upon reasonable request.
